# On the Characteristics of the Cognitive Dissonance State: Exploration Within the Pleasure Arousal Dominance Model

**DOI:** 10.5334/pb.517

**Published:** 2020-03-23

**Authors:** Alexandre Bran, David C. Vaidis

**Affiliations:** 1 Université de Paris, FR

**Keywords:** cognitive dissonance, emotion, Pleasure Arousal Dominance model, affect, hypocrisy, counter-attitudinal

## Abstract

Little is actually known about the nature and characteristics of the cognitive dissonance state. In this paper, we review the actual knowledge and the main limitations of past studies. Then, we present two studies that investigate the characteristics of the cognitive dissonance state from the perspective the Pleasure Arousal Dominance model of emotion. Study 1 (*N* = 102) used the hypocrisy paradigm and Study 2 (*N* = 130) used a counterattitudinal essay. In Study 1, participants in the Dissonance condition reported less Pleasure with each inconsistent behaviour remembered. In Study 2, participants in the Dissonance condition reported less Pleasure than participants in the Control Condition. In both studies, no significant difference was found on the Arousal and Dominance indexes. These results are among the first to link cognitive dissonance to a general model of emotions, an approach that should be pursued further.

Cognitive Dissonance Theory (Festinger, 1957) proposes that individuals experience psychological discomfort when they are confronted with inconsistent cognitions. This psychological discomfort is more generally considered to be a state of aversive arousal that can be referred as a cognitive dissonance state (CDS). For many scholars, the CDS is the core of the theory and its central process: this psychological discomfort mediates the consequences of the inconsistency exposure (Elliot & Devine, 1994; Devine, Tauer, Barron, Elliot, & Vance, 1999; Festinger, 1957; McGrath, 2017; Vaidis & Bran, 2018). Just as hunger is an aversive state driving animals to find food in order to reduce their discomfort, the CDS is considered to be an aversive state that drives humans to resolve inconsistency in order to reduce the state. However, despite its central role in the theory, the CDS has received limited consideration in the literature. Little is empirically known about its nature and characteristics and, as we will discuss below, the field may be lacking a reliable instrument to assess the CDS (Vaidis & Bran, 2019). In this vein, the current research aims to expand knowledge of the characteristics of the CDS using the framework of the Pleasure Arousal Dominance model (Mehrabian & Russell, 1974).

## Characteristics of the Cognitive Dissonance State

The CDS has been described as a state of psychological discomfort (Festinger, 1957; Elliot & Devine, 1994), a state of tension (Croyle & Cooper, 1983, Kruglanski & Shteynberg, 2012), an unpleasant feeling (Harmon-Jones, 2000a) or a state of aversive arousal (Proulx, Inzlicht, & Harmon-Jones, 2012). Most of these descriptions integrate two distinct components: arousal and negative valence. In the following sections, we review the empirical evidence linking the CDS to these two components.

### Cognitive Dissonance Arousal

The literature provides many clues that cognitive dissonance involves physiological arousal. For instance, writing a counterattitudinal essay is the predominant task used to generate an inconsistency between an attitude and a behaviour, and studies found elevated galvanic skin responses (GSR) during and after the writing of such essays (Croyle & Cooper, 1983; Elkin & Leippe, 1986; Harmon-Jones, Brehm, Greenberg, Simon & Nelson, 1996; Losch & Cacioppo, 1990). The free-choice paradigm and receiving feedback contrary to expectations are two other paradigms that have been associated with elevated heart rate (Etgen & Rosen, 1993; Gerard, 1967; Mann, Janis & Chaplin, 1969). Examinations of neuronal activity have shown a lower level of alpha waves when doing an unpleasant task without enough justification (McMillen & Geiselman, 1974) as well as the involvement of specific brain areas such as the *anterior cingulate cortex* during cognitive dissonance studies involving counterattitudinal tasks or the free-choice paradigm (for review, see Izuma & Murayama, 2019). Finally, other evidence of cognitive dissonance arousal involves the increased activity of the corrugator supercilii muscles during a counterattitudinal essay setting (Martinie, Olive, Milland, Joule, & Capa, 2013) and pupil dilation when exposed to perceptual inconsistencies (Proulx, Sleegers, & Tritt, 2017; Sleegers, Proulx, & van Beest, 2015).

Given the amount of evidence, it seems indisputable that cognitive dissonance is associated with elevated arousal. However, some deviations across these results should be noted: contrary to the results in the free-choice paradigm, studies involving writing counterattitudinal essays have not found an elevation in heart rate (Croyle & Cooper, 1983); similarly, contrary to the results for the counterattitudinal essay paradigm, studies utilizing unexpected feedback have not found an elevation in GSR (Etgen & Rosen, 1993). These discrepancies are also noticeable in neuronal studies. Although most studies report an activation of the *anterior cingulate cortex* in dissonance situations, this finding is not ubiquitous, and other discrepancies in activated areas can be seen between studies (for a review, see de Vries, Byrne, & Keho, 2015). Especially, two studies using the free-choice paradigm have not found the same activation pattern, and it is not clear if these differences are due to methodological or theoretical variations (Jarcho et al., 2010; Qin et al., 2011). Moreover, recent general criticisms of neuronal studies questioned the precision of neuronal results and it is likely that many variations between paradigms are currently missed (see Hong, Yoo, Wager, & Woo, 2019). Given these deviations, it is not clear if the elicited arousal is exactly the same or if its nature differs depending on the dissonance paradigms. Unfortunately, there is no study to our knowledge that has compared the nature of the arousal elicited by different cognitive dissonance paradigms.

### Aversiveness of Cognitive Dissonance

While most scholars agree on the notion that the CDS is aversive, this assumption has remained untested for a long time (Devine et al., 1999). Early studies on the valence of dissonance arousal mainly used the misattribution paradigm (Zanna & Cooper, 1974). In this paradigm, a plausible explanation is offered to participants to justify their discomfort. For instance, in their seminal paper, Zanna and Cooper’s participants had to ingest a placebo pill that allegedly induced a negative mood. Because of this belief, participants in the dissonance condition were inclined to misattribute their psychological discomfort to the pill instead of the inconsistency, and thus they did not show any use of an inconsistency reduction strategy. Studies using the misattribution paradigm have repeatedly shown that the CDS could be misattributed to other sources of aversive affect such as drugs (Higgins, Rhodewalt & Zanna, 1979; Zanna, Higgins, & Taves, 1976), fear of electric shock (Pittman, 1975), room lighting (Cooper, 1998) or prism goggles (Losch & Cacioppo, 1990), but also to positive sources such as cartoons (Cooper, Fazio & Rhodewalt, 1978), pleasant pictures (Drachman & Worchel, 1976) or comedy routines (Kidd & Berkowitz, 1975). If the CDS is a negative state, it is difficult to understand how individuals can misattribute it to a positive source. Incidentally, this discovery has motivated the conceptualization of the New Look Model (Cooper & Fazio, 1984) which defines cognitive dissonance as a state of neutral physiological arousal that may later be labelled positively or negatively (see also Schachter & Singer, 1962). While some data provided support for this conceptualization (e.g., Martinie et al., 2013), most scholars still consider the dissonance state to be aversive per nature.

Aside from the misattribution paradigm, the most popular method to study the CDS is the use of self-report scales. Studies have repeatedly shown that a negative affect was induced by a variety of cognitive dissonance paradigms, such as the classic counterattitudinal task paradigm (Cancino-Montecinos, Björklund, & Lindholm, 2018; Elliot & Devine, 1994; Harmon-Jones, 2000a; Galinsky, Stone, & Cooper, 2000; Shaffer, 1975), being reminded of self-transgressions concerning advocated behaviours (Pelt & Fointiat, 2018; Priolo et al., 2016; Yousaf & Gobet, 2013), being exposed to information inconsistent with beliefs (Russell & Jones, 1980; Vaidis & Gosling, 2011), being in disagreement with others (Matz & Wood, 2005), and seeing someone performing a counterattitudinal behaviour (i.e., vicarious dissonance; Norton, Monin, Cooper, & Hogg, 2003; Monin, Norton, Cooper, & Hogg, 2004). Most scholars using self-report scales consider that the CDS is not felt as a general negative affect but is rather experienced as a specific *psychological discomfort* (Elliot & Devine, 1994).

Apart from the misattribution paradigm and self-report scales, further evidence of the aversive nature of the CDS involves the activation of facial areas related to negative emotions while writing a counterattitudinal essay (Martinie et al., 2013). Overall, these results seem to show that cognitive dissonance is associated with negative affect, and most cognitive dissonance scholars conclude so. However, we believe that there are a number of important limitations to these studies.

### Limits on the Evidences for Cognitive Dissonance Aversiveness

We are mainly concerned with three limitations that call into question the validity of the studies reviewed above as evidence of the aversive nature of dissonance. First, it is unclear if the emotion captured in these studies is really the theorized CDS or a confound with other negative emotions. This is especially the case when the assessment is only based on measures that cannot assess a specific state, such as facial activity or the “calm-tense” item used by Zanna and Cooper (1974). This is problematic because many cognitive dissonance paradigms are likely to induce negative emotions other than the CDS. For instance, paradigms that potentially highlight inconsistencies with the self, such as the induced hypocrisy paradigm or counter-attitudinal tasks, are likely to induce *guilt* or *shame* in the participants (Elliot & Devine, 1994; Kenworthy et al., 2011; Stice, 1992; Yousaf & Gobet, 2013). Consequently, when reviewing the main cognitive dissonance paradigms, Kenworthy et al. (2011) conclude that *guilt* could be the most important mediator of common cognitive dissonance effects. Depending on the paradigms used, anger and surprise are two other emotions that are likely to be confounded with cognitive dissonance (Geschwender, 1967; Noordewier & Breugelmans, 2013). Some scholars would probably disregard this point by considering that *guilt* or *anger* are forms of the CDS (Geschwender, 1967; Stice, 1992); however, most seem to consider the CDS to be a specific state, distinct from these other emotions (Elliot & Devine, 1994; Kenworthy et al., 2011). While this particular debate is beyond the scope of this article, we invite consideration of the question. If the CDS is a specific state, then instruments should allow the CDS to be distinguished from other negative emotions.

A second limitation of previous studies is that there is very little evidence supporting the view that the assessed negative affect is the same across all paradigms. Most studies have focused on a restricted set of cognitive dissonance paradigms, especially on the counterattitudinal paradigm. In comparison, some other paradigms, such as the free-choice or the effort justification paradigms, have very few measures of the level of psychological discomfort they induce. This amounts to many data supporting the idea that writing a counterattitudinal essay evokes negative affect, but scarce evidence that the other paradigms induce the same negative affect. Actually, it seems likely that different inconsistencies would elicit different affective states, for instance depending on whether they involve self-relevant cognitions (Elliot & Devine, 1994) or positive outcomes for the individual (Gawronsky & Branon, 2019; Kruglanski & Shteynberg, 2012). As presented above, studies assessing arousal are unclear regarding whether the different cognitive dissonance paradigms elicit the same sort of arousal. Therefore, it is still to be determined if there exists a common CDS across these paradigms.

The third limitation is that there are few studies using reliable measures of the CDS. As we wrote above, instruments that focus on general feelings or emotions may in fact capture other emotions. As most cognitive dissonance paradigms are likely to induce other emotions, it may be more pertinent to distinctively assess the nature of the psychological discomfort involved in the CDS. In this regard, the *Dissonance Thermometer* (Devine et al., 1999; Elliot & Devine, 1994) is the most common means to assess CDS. In its original form, it is a 18 item self-report affective scale that computes a specific CDS index on the basis of how much people report feeling *uncomfortable, uneasy* and *bothered*. Today, most studies assessing the CDS rely on this instrument. However, despite its popularity, this scale seems to present several flaws. The few studies that have reported its factorial structure have found irregularities (Elliot & Devine, 1994; Gosling, Denizeau, & Oberlé, 2006; Matz & Wood, 2005), although part of this may be due to different choices of factor analysis such as the applied rotation. The scale indexes have presented an insufficient homogeneity several times (Harmon-Jones, 2000a; Priolo et al., 2016), and their inter-correlations fluctuate (Elliot & Devine, 1994; Galinsky et al., 2000; Matz & Wood 2005). Sometimes the cognitive dissonance induction has no significant effect on the identified discomfort index but affects the negative-self index (Gosling et al., 2006). Moreover, the discomfort index may also lack sensitivity, as its scores are usually very close to the lowest possible value, indicating a likely floor effect.

Perhaps due to these psychometric issues, the Dissonance Thermometer is also not used in a standardized way. The discomfort index is often assessed separately from the rest of the scale (Harmon-Jones, 2000b; Galinski, et al., 2000; Monin et al., 2004; Norton et al., 2003; Vaidis & Gosling, 2011), or measured with different instructions and methods of scoring (Harmon-Jones, 2000b; Monin et al., 2004; Norton et al., 2003; Vaidis & Gosling, 2011), and some researchers alter the index by using only some of the original items (Holland, Meertens & Van Vugt, 2002) or by combining it with other items (Jordens & Van Overwalle, 2005; Matz & Wood, 2005; Pelt & Fointiat, 2018; Priolo et al., 2016). This lack of standardization impairs the comparability of the results and limits their interpretation. For instance, could the affect assessed with Elliot and Devine’s three items (1994; *uneasy, uncomfortable* and *bothered*) and Matz and Wood’s five items (2005; *uneasy, uncomfortable, bothered, tense* and *concerned*) be considered the same? The Dissonance Thermometer has been initially used to support the claim that CDS is experienced as a specific psychological discomfort instead of a general negative affect (Elliot & Devine, 1994). However, all the variations we have seen could actually be interpreted as evidence for a general and unspecified negative affect.

In our opinion, developing an instrument assessing a specific affect, such as the CDS, requires understanding the precise nature and characteristics of this affect. However, the characteristics of the CDS are not well-understood today, aside from a general consensus that it should involve a form of negative arousal. For the purpose of developing a relevant instrument, we believe that there is much to gain from taking a step back and examining the characteristics of the CDS in a more global framework. While cognitive dissonance theory has rarely been linked to psychological models of emotions, these models can be used to better describe the nature of the CDS. Indeed, decades of research have investigated the nature and structure of human emotions, and it seems senseless to ignore this work in the examination of the CDS. In the following studies, we use the framework of the Pleasure Arousal Dominance model of emotion (Bakker, van der Voordt, Vink, & de Boon, 2014; Mehrabian & Russell, 1974) to investigate the characteristics of the CDS across two cognitive dissonance paradigms.

### An Overview of the Pleasure Arousal Dominance Model of Emotion

The Pleasure Arousal Dominance (PAD) model was conceptualized by Mehrabian and Russell (1974) to understand the characteristics of internal emotional states, and is often used to measure the impact of stimuli or environmental features on affective states (e.g., Bradley & Lang, 2000; Lang, Bradley, & Cuthbert, 2008). For instance, it has been used to categorize the emotions induced by the pictures from the International Affective Picture System (Lang et al., 2008), as well as in a vast array of research including neuropsychology, computer science, marketing research and environmental psychology (see Bakker et al., 2014).

The PAD model proposes a categorization of emotions on three independent dimensions: *pleasure, arousal* and *dominance. Pleasure* refers to the general positive and negative feelings experienced, *arousal* refers to the level of alertness and physical activity, and *dominance* refers to the feelings of control, non-restriction and autonomy. This three-dimensional structure allows comparing the characteristics of different emotional states. For instance, anger is reported as an unpleasant, aroused and moderately dominant emotion, while boredom is reported as a slightly unpleasant, unaroused and mostly non-dominant emotion (Mehrabian, 1980). Compared to the dissonance thermometer, using the PAD model may give us additional information about the characteristics of the CDS. The dimensions of pleasure and arousal may adequately capture its supposed “aversive arousal” nature. For its part, the dimension of dominance may be an interesting exploratory measure given that the CDS has similar properties to anger and is linked to an action motivation that may increase feelings of dominance (Harmon-Jones, 2000a, 2004; Harmon-Jones & Harmon-Jones, 2019; Jonas et al., 2014). However, it is also often linked to negative emotions such as guilt or shame (Kenworthy et al., 2011; Stice, 1992) that may reduce feelings of dominance. Therefore the PAD model could help in identifying the characteristics of the CDS and its distinctiveness in comparison to other emotions. In turn, knowing the characteristics of the CDS will facilitate the development of a specific measure instrument for its assessment.

Two main instruments have been developed to assess emotions using the PAD model. The first one is the PAD scale developed by Mehrabian and Russell (1974). With this scale, each of the three dimensions is measured with six bipolar items. Participants rate their affective state using pairs of items such as ‘annoyed-pleased,’ ‘relaxed-stimulated,’ or ‘controlled-controlling.’ The second instrument is the Self-Assessment Manikin scale (SAM, Bradley & Lang, 1994), a non-verbal scale that uses humanoid figures with various expressions to represent the dimensions. When used together, both the PAD scale and the SAM tend to show similar results (Bradley & Lang, 1994). To our knowledge, cognitive dissonance theory has rarely been linked with general models of emotion before, and never with the PAD model. Yet, this model appears to be a relevant framework to examine the characteristics of the CDS.

### Studies Overview

In the two studies presented below, we aimed to investigate the characteristics of the CDS by using the PAD model of emotion. We used two of the most popular paradigms in the field: the hypocrisy paradigm (Study 1) and the counterattitudinal essay paradigm (Study 2). Our main hypotheses were that in cognitive dissonance conditions, participants would report lower scores on the Pleasure index and higher scores on the Arousal index compared to the control conditions. We did not have specific expectancies for the Dominance index. Both studies were pre-registered following recommendations from van’t Veer and Giner-Sorolla’s (2016) with exact procedure, specific predictions and analysis plan. Pre-registrations, materials and data are available on Open Science Framework (Study 1: https://osf.io/q45r6; Study 2: https://osf.io/hu25f).

## Study 1: Hypocrisy Paradigm

### Participants

Because, to our knowledge, no previous study has ever assessed the CDS using the PAD scale, we chose to use the sample size recommended for achieving a .80 power to detect a medium effect size (*d* = 0.5) with a one-tailed t-test, that is, a sample size of 102 participants. We recruited 102 students in a French university in exchange for course credit. All participants were welcomed individually into a lab room by a male experimenter for a study presented as a combination of several psychology studies. Participants were randomly assigned to one of the two conditions of a between-participant experimental design (Hypocrisy vs. Control).

### Procedure

The hypocrisy paradigm consists of inducing participants to support a norm and then remembering instances of their behaviours that violated the norm (Aronson, 1992; Priolo et al., 2019). In our study, participants were first presented with a list of seven anonymous quotations aiming to increase public concern about the protection of the environment and allegedly coming from public personalities. This procedure was intended to increase the salience of the environmental protection norm (Stone & Fernandez, 2008). Participants were then instructed to write a short essay in favour of the protection of the environment. It was mentioned that their essay could be as long as they wished and that they could inspire themselves with the provided list of quotations if needed. Participants had no time limit to write their essay and it was emphasized that the content of their essay was the main focus of the research.

Once they finished their essay, participants had to complete a recall task in 2 steps. In the Hypocrisy condition, participants were first asked to take some time to privately remember all their behaviours of the last month that may have been harmful (vs. beneficial in the Control condition) to the environment. In a second step, they were shown a list of seven specific harmful behaviours concerning the environment and had to mark the ones they personally did in the last month (e.g., “Using a car for a short trip”). In the Control condition, the first step was to remember behaviour that had a positive impact on the environment and the recall task consisted of listing positive behaviours (e.g., “Not using a car for a short trip despite having the opportunity”). To ensure that participants remembered specific occurrences, we asked them to precisely state the spatial and temporal context in which each behaviour occurred. The combination of environmental protection norm salience and the recall of environmentally harmful behaviours should induce cognitive dissonance (Priolo et al., 2016).

Right after the recall phase, participants completed the French validated version of the PAD scale (Detandt, Leys, & Bazan, 2017). This self-report scale is composed of 18 items that assess three main dimensions: Pleasure, Arousal and Dominance. Participants are asked to report how they feel at that moment on a 9-point bipolar scale from –4 (e.g., *pleased*) to +4 (e.g., *annoyed*). After the affect measures, participants completed two exploratory tasks, were debriefed and thanked.

### Results

It was hypothesized that participants in the Hypocrisy condition would experience less Pleasure and more Arousal than participants in the Control condition. No hypothesis was made for the Dominance index.

#### Pre-registered analyses

The PAD scale presented adequate internal consistency on the Pleasure (α = .81), Arousal (α = .74) and Dominance (α = .76) indexes.

A one-way MANOVA showed a marginally significant main effect of the experimental conditions on the PAD scale: *F*(3, 99) = 2.58, *p* = .06, Wilk’s Λ = 0.93, partial η^2^ = .07, *d* = 0.56; with notably less positive affect and more arousal. Sensitivity analyses show that we achieved a .64 power to detect such effect size. Univariate analyses did not confirm significant differences for the three components: Pleasure (*F*(1, 100) = 0.89; *p* = .35), Arousal (*F*(1, 100) = 1.61; *p* = .21) and Dominance (*F*(1, 100) = 1.56; *p* = .22) (see Table 1).

**Table 1:** Means Comparisons Across Dependent Variables of Study 1.

Variable	Hypocrisy	Control	95% CI of difference

Pleasure	1.98 (0.99)	2.19 (1.22)	[–0.65; 0.23]
Arousal	1.31 (1.21)	1.02 (1.13)	[–0.16; 0.75]
Dominance	1.19 (1.16)	1.45 (0.95)	[–0.68; 0.16]

*Notes*: Higher scores indicate respectively more pleasure, more arousal and more dominance. Standard deviations are in parentheses.

#### Complementary analyses

We conducted a principal component analysis of the PAD scale with an OBLIMIN rotation. The analysis confirmed the 3-factor structure of the scale. All items loaded accordingly to their index, except for two items of the dominance index that did not load strongly on any of the components (*important/awed* and *in control/cared for*). As this analysis was not pre-registered, we decided to keep the latter items in the analyses.

Although participants were all instructed to recall seven specific occurrences of behaviours, not all did so, and we observed variations in the number of reported behaviours (*M* = 4.35; *SD* = 1.30). Because participants reporting seven inconsistent behaviours may have experienced more cognitive dissonance than participants reporting, for example, only two (Fointiat, Morisot, & Pakuszewski, 2008; Kruglanski et al., 2018), we decided to take into consideration the number of behaviours in our analyses.

**Figure 1:**
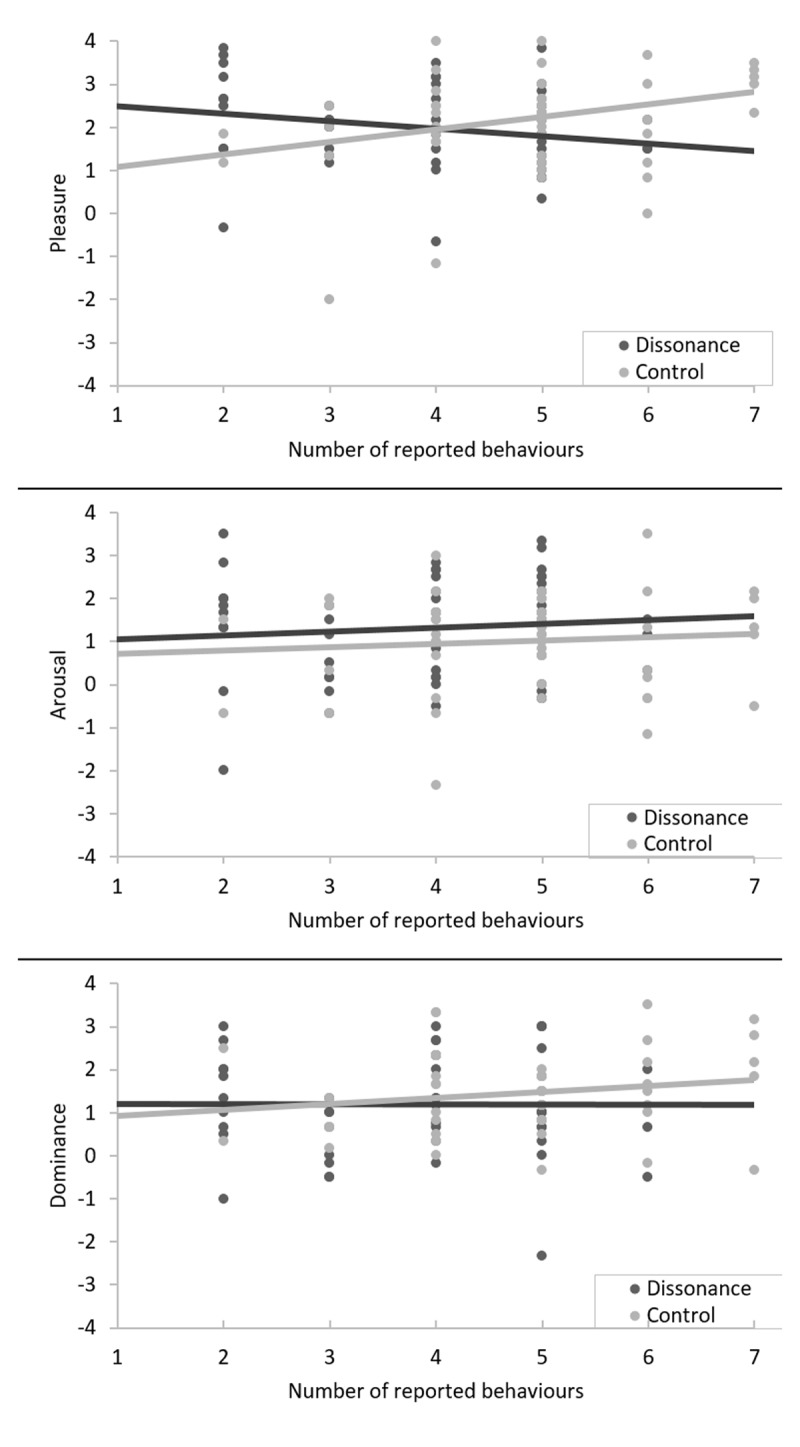
Interaction between the number of reported behaviours and the experimental condition on reports of Pleasure, Arousal and Dominance.

Linear regression analyses were performed that included the effect of condition (Control vs Hypocrisy), the number of reported behaviours, and their interaction term in order to predict the emotion scores. Supporting the notion of psychological discomfort, we found that participants in the Hypocrisy condition experienced less *pleasure* with each behaviour recalled, inversely to participants in the Control condition (see Figure 1). Full model: *F*(3, 98) = 2.75, *p* = .05, R^2^ =.08, *d* = 0.58; Pleasure: *B* = –0.46, *t*(98) = –2.64, *p* = .01, R^2^ = .065 *d* = 0.52. No significant effects were observed on *arousal* and *dominance* scores (both *ps* > .41).

## Study 2: Counterattitudinal Essay

### Participants

Study 1 main analysis resulted in a *d* of 0.56. For study 2, we chose to base our power analysis on a two-tailed test. This aimed both to increase our sample size and to allow for the examination of contradictory hypotheses on the PAD scores. We planned to recruit at least the necessary number of participants to achieve a .80 power to detect a *d of* 0.50 with a two-tailed *t*-test, that is 128 participants. A total of 143 students participated in the study in exchange for course credit. Among them, 13 refused to write the requested essay (5 Pro; 8 Counter) and were discarded from the analyses. The final sample consisted of 130 participants (*M*_age_ = 20; *SD*_age_ = 4.15; 104 women and 1 unreported). Participants were randomly assigned to the experimental conditions.

### Procedure

Participants were invited to participate in a study about students’ attitudes toward tuition fees. They read instructions explaining that a faculty committee wanted to know students’ attitudes towards a possible increase in tuition fees. In order to know the general attitudes of the students, participants were told that they would have to write an essay either in favour of or against an increase in tuition fees. In the Counter-attitudinal condition, all participants were told that they would have to write an essay in favour of an increase in inscription fees. In the Pro-attitudinal condition, participants could freely choose to write either in favour or opposing an increase. In this condition, only seven participants (11%) chose to write in favour of an increase, thus confirming the idea that writing arguments in favour of an increase was counterattitudinal for most students.

In both conditions, we used commitment variables to maximize the magnitude of cognitive dissonance in the Counter-attitudinal condition (i.e., aversive consequence, freedom, publicity; see Harmon-Jones & Mills, 1999; Kiesler, 1971). The publicity and the consequences of the act were high, as participants were instructed to sign the consent form with their name and were told that their arguments would be presented to the committee. Free-choice was emphasized by telling participants that they were free to participate or not in the study and that they could quit the study at any time, without any loss of benefits or other negative consequences.

Once participants signed the consent form, they began writing their essay. After 1 minute of writing, the experimenter feigned to have forgotten a phase of the study and gave the PAD scale to the participants. The scale was the same as in Study 1 and its instructions explained the need to assess the participants’ emotions, as they could influence their essay. At the end of the scale, a complementary question assessed participants’ attitude towards an increase of inscription fees on a 7-point-scale ranging from 1 (*totally disagree*) to 7 (totally agree). Finally, participants were debriefed and thanked.

### Results

It was hypothesized that participants in the Counter-attitudinal condition would experience less Pleasure and more Arousal than participants in the Pro-attitudinal condition. As a classic result in dissonance studies, they were also expected to report more positive attitudes toward the counterattitudinal topic.

#### Pre-registered analyses

The PAD scale presented adequate internal consistency on the Pleasure (α = .87) and Arousal (α = .76) indexes, and low internal consistency on the Dominance index (α = .54). A one-way MANOVA showed a main effect on the PAD scale: *F*(3, 126) = 4.63; *p* = .004, Wilk’s Λ = 0.90, partial η^2^ = .10, *d* = 0.66. Sensitivity analyses show that we achieved a .89 power to detect such effect size. Univariate analyses showed that participants in the Counter-attitudinal condition reported significantly less Pleasure than in the Pro-attitudinal condition: *F*(1,128) = 13.49, *p* < .001, partial η^2^ = .10, *d* = 0.65 (see Table 2). No significant differences were observed for Arousal (*p* = .68) and Dominance (*p* = .21) scores.

**Table 2:** Means Comparisons Across Dependent Variables of Study 2.

Variable	Counterattitudinal	Proattitudinal	95% CI of difference

Pleasure	1.87 (1.16)	2.59 (1.06)	[–1.00; –0.29]
Arousal	1.03 (1.04)	1.01 (1.19)	[–0.42; 0.27]
Dominance	1.47 (0.73)	1.63 (0.71)	[–0.57; 0.12]
Attitude	2.88 (1.24)	2.27 (1.06)	[0.21; 1.01]

*Notes*: Higher scores respectively indicate more pleasure, more arousal, more dominance and more positive attitude. Standard deviations are in parentheses.

As expected, we observed a classic dissonance effect: *t*(127) = 3.01, *p* = .003, *d* = 0.53. Participants in the Counter-attitudinal condition were significantly more favourable to an increase in tuition fees (*M* = 2.88; *SD* = 1.24) than participants in the Pro-attitudinal condition (*M* = 2.27; *SD* = 1.06).

#### Complementary analyses

We conducted a principal component analysis of the PAD scale. An OBLIMIN rotation resulted in a three factors structure. Almost all items loaded accordingly to their respective components except one item of Pleasure that loaded on Arousal (*relaxed* – *bored*) and one item of Dominance that did not load substantially on any component (*important* – *awed*). As this analysis was not pre-registered, we decided to use the latter items in the main analyses.

It is also theoretically assumed that the CDS mediates regulations strategies, such that the stronger the CDS is, the stronger the regulation should be (Festinger, 1957; Sakai, 1999). However there is currently mixed support in the literature for this proposition, with a number of unsupporting results (Harmon-Jones, 2000b; Norton, Monin, Cooper & Hogg, 2003, study 3; Rhodewalt & Comer, 1979; Simmons & Brandon, 2007). Therefore, while this was not one of this study’s objectives, we performed a linear regression analysis to test the effect of Pleasure score and of condition (Pro-attitudinal vs Counter-attitudinal) on attitude score: *F*(3, 126) = 5.01, *p* = .01, R^2^ = .07, *d* = 0.56. Participants in the Counter-attitudinal condition reported more favourable attitude than participants in the Pro-attitudinal condition: *B* = 0.68, *t*(126) = 3.16, *p* < .01, R^2^ = .07. Contrary to the mediation hypothesis, pleasure did not significantly predict attitude score: *B* = 0.09, *t*(126) = 0.99, *p* = .32, R^2^ = .01.

## Discussion

In two studies, we aimed to examine the CDS within the framework of the PAD model. We used two of the most popular paradigms to assess how the CDS impacts the Pleasure, Arousal and Dominance indexes of the PAD scale. The present results provide information with regard to our understanding of the CDS and are partially satisfying concerning the use of the PAD scale to assess the CDS.

Concerning the valence of CDS, in Study 1, significant differences were obtained on the PAD scores, although we did not obtain the anticipated significant results for each PAD dimension separately. However, post-hoc analyses revealed that participants in the Hypocrisy condition reported less Pleasure with each behaviour remembered, which might indicate an increased dissonance state, contrary to participants in the Control Condition. In Study 2, participants in the Counter-attitudinal condition reported less Pleasure than participants in the Pro-attitudinal condition. These two results support that cognitive dissonance induces an unpleasant state and that this quality can be captured by the Pleasure dimension of the PAD scale. Interestingly, our results are among the few that link cognitive dissonance to a general and non-specific negative affect (see also Harmon-Jones, 1999, Study 1). Most studies in the literature associate the CDS to a specific index (e.g., Elliot & Devine, 1994) and do not find a significant link between cognitive dissonance and general negative affect. However, as we discussed previously, most of these studies used the Dissonance Thermometer, which may be unsuited for the assessment of general negative affect. In this regard, comparisons of negative affect assessed by the two instruments could be explored in future studies. If the CDS is experienced as a general negative affect instead of a specific one, than it could be experienced and reported differently depending on the context. For instance, violating a supported norm could evoke a general negative affect that would be interpreted and reported as guilt (Harmon-Jones, Harmon-Jones, & Summerell, 2017). In this case, this guilt would be evidence of cognitive dissonance (see for instance Yousaf & Gobet, 2013).

Concerning arousal, contrary to our hypothesis, neither Study 1 nor Study 2 have shown a significant effect of the CDS on participants’ reports of arousal. Given the strength of supporting evidence linking cognitive dissonance to arousal in the literature, this absence is peculiar. The successful detection of increased negative valence in both studies, as well as attitude change in Study 2, make us believe that the inductions should have been sufficient to evoke arousal. A likely explanation is that the PAD scale was not sensitive enough to capture the arousal properties of the CDS in our studies. It is also possible that participants are not aware of their internal arousal state and therefore have difficulty precisely reporting their level of arousal (Scheier, Carver, & Gibbons, 1979), thus making self-report scales less efficient in this regard than physiological assessments.

To our knowledge, our studies are the first to assess the consequences of cognitive dissonance on feeling of dominance. While not part of our primary hypothesis, there are several reasons to consider that dominance can be linked to cognitive dissonance, some of them suggesting contradictory hypotheses, such as the links between cognitive dissonance, anger (Geschwender, 1967) and shame (Stice, 1992), or the activation of an action motivation (Harmon-Jones & Harmon-Jones, 2019). These contradictory hypotheses have not been investigated yet in the literature, and our studies, while inconclusive, report the first available data on these questions. However we find that the internal consistency of the Dominance index was questionable in our studies. Researchers using the PAD scale often report that the Dominance index is less internally consistent than the Pleasure and Arousal indexes (e.g., Broekens, 2012). It is possible that using the non-verbal scales associated with the PAD model would help circumventing this issue and allow further exploration of the links between the CDS and feelings of dominance (i.e., SAM, Bradley & Lang, 1994; Affect Button, Broekens, 2012). Another possibility would be to use more specific instruments, such as those constructed to assess sense of agency or sense of control (e.g., Tapal, Oren, Dar & Eitam, 2017).

Finally, an interesting pattern in Study 1 should be discussed: participants who remembered only a few inconsistent behaviours in the Hypocrisy condition tended to report more Pleasure than participants who reported only a few consistent behaviours in the Control condition. Interestingly, participants who see themselves unable to report several occurrences of positive behaviours may also experience some form of dissonance, as they see themselves as not doing enough for the environment. Moreover, participants who only remember one or two inconsistent behaviours can reassure themselves when seeing that they are not concerned by all the other behaviours, and may thus consider that they are globally doing fine. These results are interesting because they can be linked to related hypotheses about the magnitude of the dissonance. In the vein of Festinger’s original statement (1957), several scholars consider that the more numerous or important the cognitions involved in the cognitive dissonance state are, the stronger it should be (Kruglanski et al., 2018; Vaidis & Bran, 2018). That is a hypothesis of a linear relation between inconsistency and magnitude of dissonance. To our knowledge, no study has yet fully demonstrated the function relation between inconsistency, CDS and the regulation process, mostly because there are few studies that include variation in the degree of inconsistency. While it was not its prime objective, Study 1 provides interesting clues as to how to test this hypothesis with the hypocrisy paradigm.

## Conclusion

The present paper aimed to investigate the nature of the CDS. In two pre-registered studies, we investigated the usefulness of the PAD model to assess the CDS induced by the hypocrisy paradigm (Study 1) and by writing a counterattitudinal essay (Study 2). Combined together, our two studies show that the CDS induced in the hypocrisy and counterattitudinal paradigms is first and foremost characterised by increased negative valence, as captured presently by the Pleasure dimension. Our studies did not find evidence of a role for Arousal or Dominance change, which suggest that these characteristic are less defining features of the dissonance state. While the sensitivity of the PAD scale seems moderate in regards to our results, we believe that cognitive dissonance scholars should continue to reconnect with general models of emotion to investigate the CDS. It is only by understanding the precise nature of the CDS that the field will be able to construct an internally consistent instrument for its assessment, a subject that has motivated more and more research in recent times (e.g., Levy, Harmon-Jones & Harmon-Jones, 2017). In our opinion, these studies are valuable as the psychological discomfort is supposed to be the core of the theory and the mediator of all cognitive dissonance. In this context, further studies investigating the nature and characteristics of the CDS will be informative and will help understanding the processes behind cognitive dissonance.
